# Association of *BRAF* Variants With Disease Characteristics, Prognosis, and Targeted Therapy Response in Intrahepatic Cholangiocarcinoma

**DOI:** 10.1001/jamanetworkopen.2023.1476

**Published:** 2023-03-03

**Authors:** Hao-Yang Xin, Rong-Qi Sun, Ji-Xue Zou, Peng-Cheng Wang, Jia-Yin Wang, Yu-Hang Ye, Kai-Xuan Liu, Zhi-Qiang Hu, Zheng-Jun Zhou, Jia Fan, Jian Zhou, Shao-Lai Zhou

**Affiliations:** 1 Department of Liver Surgery and Transplantation, Zhongshan Hospital, Fudan University, Shanghai, China; 2 Liver Cancer Institute, Zhongshan Hospital, Fudan University, Shanghai, China

## Abstract

**Importance:**

*BRAF* variants are associated with tumor progression; however, the prevalence of *BRAF* variant subtypes and their association with disease characteristics, prognosis, and targeted therapy response in patients with intrahepatic cholangiocarcinoma (ICC) are largely unknown.

**Objective:**

To explore the association of *BRAF* variant subtypes with disease characteristics, prognosis, and targeted therapy response in patients with ICC.

**Design, Setting, and Participants:**

In this cohort study, 1175 patients who underwent curative resection for ICC from January 1, 2009, through December 31, 2017, were evaluated at a single hospital in China. Whole-exome sequencing, targeted sequencing, and Sanger sequencing were performed to identify *BRAF* variants. The Kaplan-Meier method and log-rank test were used to compare overall survival (OS) and disease-free survival (DFS). Univariate and multivariate analyses were performed using Cox proportional hazards regression. Associations between *BRAF* variants and targeted therapy response were tested in 6 *BRAF*-variant, patient-derived organoid lines and in 3 of the patient donors of those lines. Data were analyzed from June 1, 2021, to March 15, 2022.

**Interventions:**

Hepatectomy in patients with ICC.

**Main Outcomes and Measures:**

The association of *BRAF* variant subtypes with OS and DFS.

**Results:**

Of 1175 patients with ICC, the mean (SD) age was 59.4 (10.4) years and 701 (59.7%) were men. A total of 20 different subtypes of *BRAF* somatic variance affecting 49 patients (4.2%) were identified; V600E was the most frequent allele in this cohort, accounting for 27% of the identified *BRAF* variants, followed by K601E (14%), D594G (12%), and N581S (6%). Compared with patients with non-V600E *BRAF* variants, patients with *BRAF* V600E variants were more likely to have large tumor size (10 of 13 [77%] vs 12 of 36 [33%]; *P* = .007), multiple tumors (7 of 13 [54%] vs 8 of 36 [22%]; *P* = .04), and more vascular/bile duct invasion (7 of 13 [54%] vs 8 of 36 [22%]; *P* = .04). Multivariate analysis revealed that *BRAF* V600E variants, but not overall *BRAF* variants or non-V600E *BRAF* variants, were associated with poor OS (hazard ratio [HR], 1.87; 95% CI, 1.05-3.33; *P* = .03) and DFS (HR, 1.66; 95% CI, 1.03-2.97; *P* = .04). There were also broad differences among organoids with different *BRAF* variant subtypes in sensitivity to BRAF or MEK inhibitors.

**Conclusions and Relevance:**

The findings of this cohort study suggest that there are broad differences among organoids with different *BRAF* variant subtypes in sensitivity to BRAF or MEK inhibitors. Identifying and classifying *BRAF* variants may be able to help guide precise treatment for patients with ICC.

## Key Points

**Question:**

What is the prevalence of *BRAF* variant subtypes and their association with disease characteristics, prognosis, and targeted therapy response in patients with intrahepatic cholangiocarcinoma?

**Findings:**

In this cohort study including 1175 patients, a total of 20 different subtypes of *BRAF* somatic variants affecting 49 patients were identified, including V600E (27%), K601E (14%), D594G (12%), and N581S (6%). Patients with *BRAF* V600E variants were more likely to have larger tumor size, multiple tumors and more vascular/bile duct invasion.

**Meaning:**

The findings of this cohort study suggest that there are broad differences among organoids with different *BRAF* variant subtypes in sensitivity to BRAF or MEK inhibitors.

## Teaser

This cohort study examines *BRAF* variant subtypes, classifying them and analyzing response to therapy in patients with intrahepatic cholangiocarcinoma.

## Introduction

Intrahepatic cholangiocarcinoma (ICC) is the second most common primary liver cancer and has an increasing incidence worldwide, accounting for 20% of all cholangiocarcinomas.^1,2^ Characterized by high invasiveness and frequent postoperative recurrence, ICC exhibits one of the highest mortality rates among human cancers.^1,3^ Despite recent progress in understanding ICC pathogenesis and novel therapies with specific targets, such as *IDH1* variance^4^ and *FGFR2* fusion,^5^ ICC is still incurable in most cases.^6,7,8,9^

*BRAF* is a member of the rapidly accelerated fibrosarcoma (RAF) kinase family of mammalian cytosolic serine/threonine kinases and transduces signals downstream of rat sarcoma (RAS) via the mitogen-activated protein kinase (MAPK) pathway.^10^ *BRAF* is among the most commonly altered kinases in human cancer, and particularly melanoma, in which nearly half of tumors harbor an activating variance in *BRAF*.^11^ *BRAF* is also altered in 10% to 70% of thyroid cancers,^12^ approximately 10% of colorectal cancers (CRCs),^13^ and 3% to 5% of non–small cell lung cancer (NSCLC).^14^ Among all types of *BRAF* variants, the hotspot V600E/K variant is the most frequent allele and has been well characterized. In addition, non-V600 *BRAF* variants have been identified in many types of cancer. In certain tumor types, non-V600 variants are even more prevalent than V600 variants. For example, approximately 50% to 80% of *BRAF* variants in NSCLC and 22% to 30% of *BRAF* variants in CRC are non-V600 variants.^10^

Some reports have proposed a 3-group classification scheme for *BRAF* variants based on data that include the ability of *BRAF* variants to signal as monomers or dimers and their dependency on RAS signaling.^15,16^ Class 1 *BRAF* variants affect amino acid V600 and function as RAS independent active monomers. Class 2 variants function as RAS independent active dimers. Class 3 variants are kinase impaired but increase MAPK pathway signaling through enhanced RAS binding and subsequent CRAF activation. These distinct classes of *BRAF* variants predict responses to targeted therapies and have important implications for drug development.^10^ The prevalence of class 1, 2, and 3 variance differs widely by cancer type. Class 2 vs 3 variants are noted in melanoma (11.3% vs 9.4%), CRC (5.6% vs 15.2%), and NSCLC (20.8% vs 19.1%), which commonly carry *BRAF* variants.^10^

To our knowledge, the distribution of *BRAF* variant subtypes and classes in ICC has not previously been investigated. Their association with disease characteristics and patient prognosis is unclear as well. Therefore, we investigated the prevalence of *BRAF* variant subtypes and their association with survival and recurrence in a large cohort of patients with ICC from China. We also explored the potential implication of the variants for estimating response to targeted therapies.

## Methods

### Patients and Follow-up

This report follows the Strengthening the Reporting of Observational Studies in Epidemiology (STROBE) reporting guideline for cohort studies. We first recruited 1291 patients with primary ICC who received curative resection from January 1, 2009, to December 31, 2017, in the Department of Liver Surgical Oncology of Zhongshan Hospital, Fudan University, Shanghai, China, and tissue samples from tumor and matched noncancerous liver samples were continually collected. Patients receiving palliative operations or prior interventions (eg, transhepatic artery embolization, chemotherapy, or radiotherapy) or with other primary cancers and inflammatory diseases during follow-up were excluded from the study. Curative resection was defined as complete resection of tumor nodules, with cancer-free tumor margins shown by histologic examination, and resection of regional lymph nodes, including the hilar, hepatoduodenal-ligament, and caval lymph nodes, with no cancerous thrombus in the portal vein (main trunk or major branches), hepatic veins, or bile duct.^17^ Patients with further lymph node involvement were considered to have distant metastasis and were excluded from the study. After excluding 116 patients for these reasons, a total of 1175 patients with primary ICC were enrolled in this study. Tumor differentiation was graded histologically according to the Edmondson-Steiner criteria.^18^ Liver function was graded according to the Child-Pugh system. Tumor stage was determined according to the 2017 International Union Against Cancer TNM system. Before the operation and tissue sample collection, we obtained oral and written informed consent from each participant, with information such as the use of tissue samples and clinical characteristics for scientific research, which was granted by the research ethics committee of Zhongshan Hospital. For this study, the research ethics committee of Zhongshan Hospital granted ethical approval for the use of humans and review and approval of this study. We also obtained oral informed consent for inclusion in the study from participants at the time of follow-up. The clinicopathologic characteristics of the patients are listed in eTable 1 in Supplement 1.

A concise flowchart of this study is shown in eFigure 1 in Supplement 1. In these ICCs, 705 cases were frozen samples and 470 cases were formalin fixation and paraffin embedding (FFPE) samples. A total of 204 ICCs with frozen samples underwent whole-exome sequencing, and 501 ICCs with frozen samples and 32 ICCs with FFPE samples underwent targeted sequencing. All *BRAF* variants identified through whole-exome sequencing and targeted sequencing were validated by Sanger sequencing. All coding exons of *BRAF* identified to harbor somatic variants were further screened in an additional 438 ICCs (FFPE samples).

The present study includes follow-up data collected through December 31, 2020. Data were analyzed from June 1, 2021, to March 15, 2022. The follow-up procedures are described in detail elsewhere.^17,19^ We diagnosed tumor recurrence on the basis of computed tomography scans, magnetic resonance imaging, digital subtraction angiography, and elevated serum carbohydrate antigen 19-9 level, with or without histologic confirmation.^17^ We defined disease-free survival (DFS) as the interval between the operation and any diagnosis of recurrence (intrahepatic or extrahepatic). We defined overall survival (OS) as the time from the date of the operation until death or the end of follow-up.^17,20^ The surviving patients were censored at the time of the end of follow-up.

### Statistical Analysis

Statistical analyses were performed in the R environment, version 4.0.5 (R Foundation for Statistical Computing) or by using SPSS, version 16.0 for Windows (IBM SPSS). The χ^2^ tests were used to compare categorical data when the sample size was greater than or equal to 40 and the theoretical frequency was T greater than or equal to 5; otherwise, Fisher exact tests were used. The Kaplan-Meier method was used to calculate the OS and DFS. Differences were analyzed by the log-rank test. Univariate and multivariate analyses were performed using the Cox proportional hazards regression model. Variables with differences that were significant in univariate analyses were subsequently evaluated in multivariate analyses using a Cox proportional hazards regression model. All tests were 2-sided, and *P* values <.05 were considered to be statistically significant. Other information is available in the eMethods in Supplement 1.

## Results

### *BRAF* Variant Subtypes and Classes

**Figure 1.**
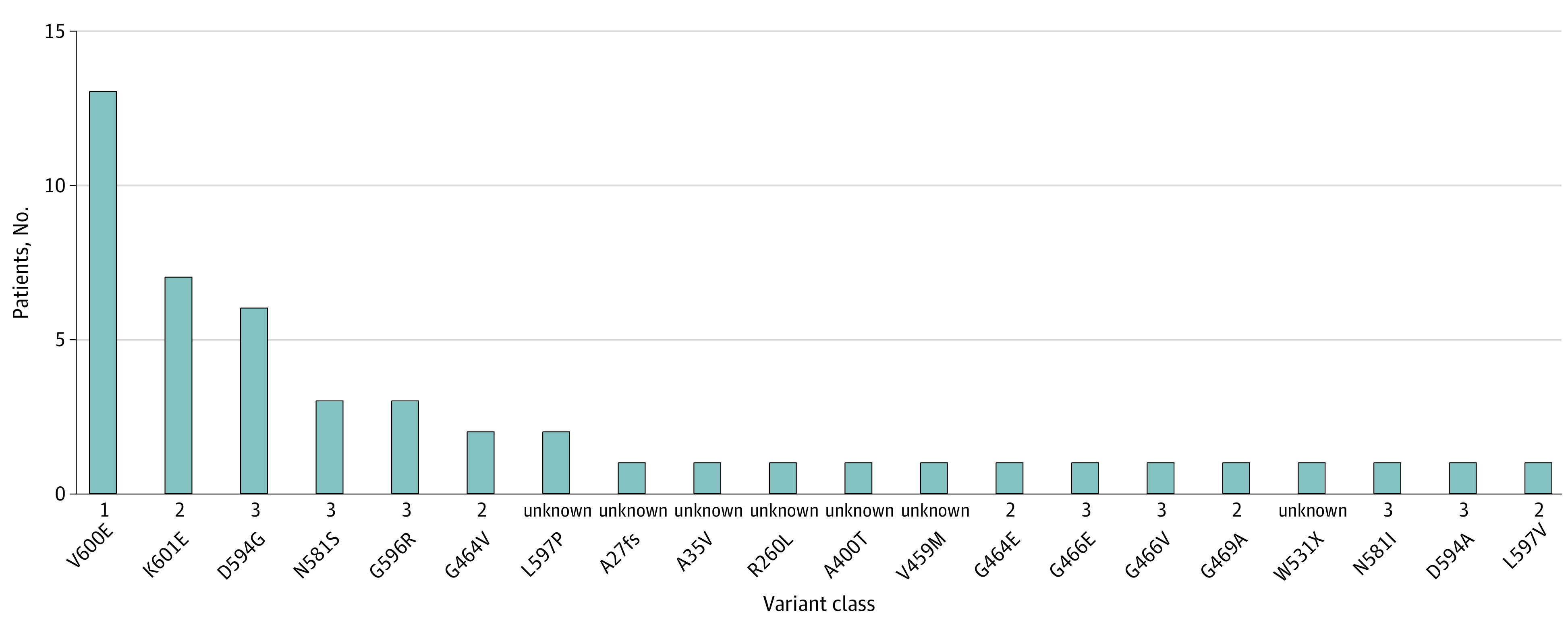
*BRAF* Variants Identified in Patients With Intrahepatic Cholangiocarcinoma Frequency of *BRAF* variant subtypes in 49 patients with *BRAF* variants with intrahepatic cholangiocarcinoma. Unknown indicates unknown function.

In 1175 patients involved in this study (701 men [59.7%] and 474 women [40.3%]; mean [SD] age, 59.4 [10.4] years), we identified a total of 20 different subtypes of *BRAF* somatic variants, which affected 49 (4.2%) patients. Thirteen patients demonstrated a V600E genotype, which was the most frequent allele in the cohort, accounting for 27% of the patients carrying *BRAF* variants. Other frequent *BRAF* alleles were K601E (7 patients [14%]), D594G (6 patients [12%]), N581S (3 patients [6%]), and G596R (3 patients [6%]) (Figure 1, eTable 2 in Supplement 1). Of the 49 patients with *BRAF* variants, 13 had class 1 *BRAF* variants (27%), 12 had class 2 *BRAF* variants (24%), 16 had class 3 *BRAF* variants (33%), and 8 had somatic *BRAF* variants of unknown function (Figure 1, eTable 2 in Supplement 1).^10,15^

### Clinical Characteristics

eTable 3 in Supplement 1 presents the clinical characteristics of the patients with wild-type *BRAF* and variant *BRAF* genotypes (including *BRAF* V600E variants and non-V600E *BRAF* variants). We observed no significant difference in clinical characteristics between the patients with wild-type *BRAF* and the patients with variant *BRAF*. Among the patients with variant *BRAF*, the patients with *BRAF* V600E variants were more likely than those with non-V600E *BRAF* variants to have large tumor size (10 of 13 [77%] vs 12 of 36 [33%]; *P* = .007), multiple tumors (7 of 13 [54%] vs 8 of 36 [22%]; *P* = .04), and more vascular/bile duct invasion (7 of 13 [54%] vs 8 of 36 [22%]; *P* = .04).

### Survival Analysis

**Figure 2.**
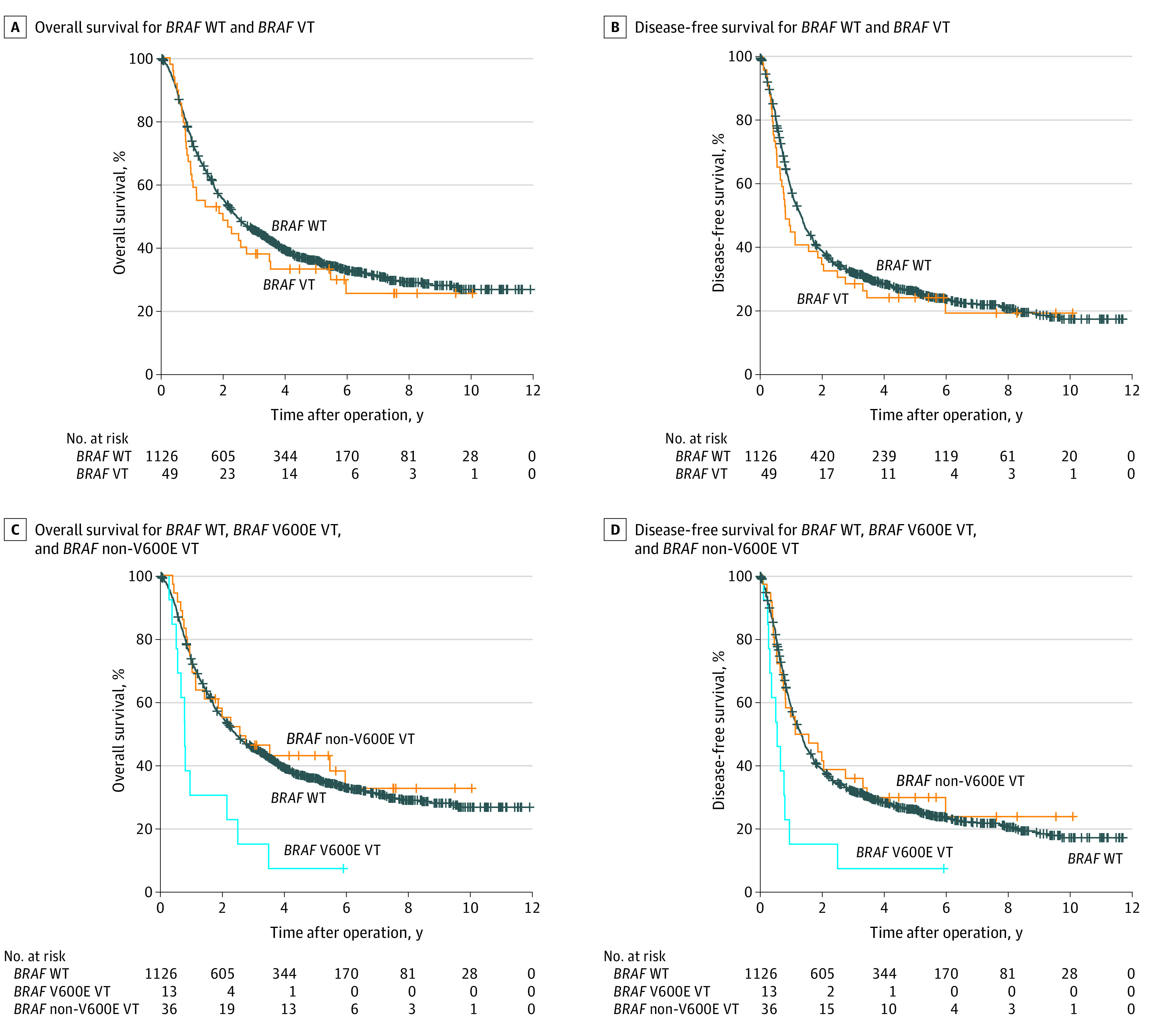
Association of *BRAF* Variants With Patient Outcome VT indicates variant; WT, wild-type.

At a median follow-up of 65.5 months (IQR, 49.1-95.3 months), 759 of the 1175 patients with ICC (64.6%) had died, and 61 patients (5.2%) were lost to follow-up. Across all patients considered, *BRAF* status was not associated with OS (hazard ratio [HR], 1.17; 95% CI, 0.83-1.65; *P* = .38) or DFS (HR, 1.15; 95% CI, 0.83-1.59; *P* = .40) (Table, Figure 2A and B). When we divided the *BRAF* variants into *BRAF* V600E and non-V600E *BRAF* subtypes, we observed that patients with *BRAF* V600E variants had significantly inferior OS (median OS, 9.1; 95% CI, 7.2-11.1 months vs 28.9; 95% CI, 25.0-32.8 months; *P* = .001) and DFS (median DFS, 6.4; 95% CI, 2.3-10.5 months vs 15.7; 95% CI, 14.2-17.3 months; *P* = .002) compared with patients with wild-type *BRAF*. The patients with non-V600E *BRAF* variants had a median OS (30.5; 95% CI, 5.2-55.8 months) and DFS (13.4; 95% CI, 0.0-28.9 months) comparable to that of patients with wild-type *BRAF* (Figure 2C and D). In the univariate analysis, *BRAF* V600E variants, but not non-V600E *BRAF* variants, were significantly associated with OS (HR, 2.52; 95% CI, 1.42-4.47; *P* = .002) and DFS (HR, 2.40; 95% CI, 1.35-4.24; *P* = .003) (Table). In multivariate analysis, *BRAF* V600E variants remained associated with poor OS (HR, 1.87; 95% CI, 1.05-3.33; *P* = .03) and DFS (HR, 1.66; 95% CI, 1.03-2.97; *P* = .04) (Table).

**Table.** Univariate and Multivariate Analyses of Prognostic Factors in 1175 Patients With Intrahepatic Cholangiocarcinoma

Variable	Univariate analyses		Multivariate analyses	
	HR (95% CI)	*P* value^a^	HR (95% CI)	*P* value^a^
**Overall survival**				
Age (>50 vs ≤50 y)	1.22 (1.02-1.47)	.03	1.29 (1.06-1.56)	.01
Sex (male vs female)	1.11 (0.96-1.28)	.17	NA	NA
HBsAg (positive vs negative)	0.69 (0.59-0.81)	<.001	0.78 (0.66-0.93)	.006
CA 19-9 (>36 vs ≤36 U/mL)	1.54 (1.33-1.79)	<.001	1.33 (1.14-1.55)	<.001
GGT (>54 vs ≤54 U/L)	1.84 (1.60-2.13)	<.001	1.43 (1.22-1.67)	<.001
Liver cirrhosis (yes vs no)	0.80 (0.67-0.95)	.01	0.94 (0.79-1.13)	.52
Tumor size (>5 vs ≤5 cm)	1.71 (1.48-1.97)	<.001	1.25 (1.07-1.47)	.005
Tumor No. (multiple vs single)	2.11 (1.81-2.47)	<.001	1.60 (1.35-1.88)	<.001
Microvascular/bile duct invasion (yes vs no)	1.66 (1.41-1.95)	<.001	1.33 (1.12-1.57)	.001
Lymphatic metastasis (yes vs no)	3.21 (2.69-3.83)	<.001	2.52 (2.10-3.04)	<.001
Tumor encapsulation (none vs complete)	1.32 (1.06-1.65)	.02	1.11 (0.88-1.40)	.37
Tumor differentiation (III+IV vs I+II)	1.40 (1.21-1.62)	<.001	1.40 (1.20-1.62)	<.001
*BRAF* (VT vs WT)	1.17 (0.83-1.65)	.38	NA	NA
*BRAF* (non-V600E VT vs WT)	0.64 (0.59-1.38)	.63	NA	NA
*BRAF* (V600E VT vs WT)	2.52 (1.42-4.47)	.002	1.87 (1.05-3.33)	.03
**Disease-free survival**				
Age (>50 vs ≤50 y)	0.96 (0.82-1.13)	.64	NA	NA
Sex (male vs female)	1.17 (1.02-1.34)	.02	1.18 (1.02-1.35)	.02
HBsAg (positive vs negative)	0.88 (0.76-1.01)	.08	NA	NA
CA 19-9 (>36 vs ≤36 U/mL)	1.46 (1.27-1.68)	<.001	1.25 (1.09-1.45)	.002
GGT (>54 vs ≤54 U/L)	1.70 (1.49-1.95)	<.001	1.39 (1.21-1.60)	<.001
Liver cirrhosis (yes vs no)	0.91 (0.78-1.07)	.25	NA	NA
Tumor size (>5 vs ≤5 cm)	1.78 (1.56-2.04)	<.001	1.38 (1.19-1.60)	<.001
Tumor No. (multiple vs single)	2.04 (1.76-2.37)	<.001	1.55 (1.32-1.81)	<.001
Microvascular/bile duct invasion (yes vs no)	1.68 (1.44-1.95)	<.001	1.31 (1.12-1.54)	.001
Lymphatic metastasis (yes vs no)	2.73 (2.30-3.24)	<.001	2.14 (1.79-2.56)	<.001
Tumor encapsulation (none vs complete)	1.12 (0.92-1.36)	.26	NA	NA
Tumor differentiation (III+IV vs I+II)	1.28 (1.11-1.46)	<.001	1.23 (1.07-1.41)	.004
*BRAF* (VT vs WT)	1.15 (0.83-1.59)	.40	NA	NA
*BRAF* (non-V600E VT vs WT)	0.93 (0.63-1.37)	.70	NA	NA
*BRAF* (V600E VT vs WT)	2.40 (1.35-4.24)	.003	1.66 (1.03-2.97)	.04

Abbreviations: CA 19-9, carbohydrate antigen 19-9; GGT, gamma-glutamyltransferase; HBsAg, hepatitis B surface antigen; HR, hazard ratio; NA, not applicable; VT, variant; WT, wild-type.

SI conversion factor: To convert GGT to microkatals per liter, multiply by 0.0167.

^a^ *P* values determined using a Cox proportional hazards regression model.

We further noted the association between *BRAF* variants and OS using genomic data of 514 ICC samples from 2 studies in cBioPortal.^8,21,22^ Kaplan-Meier analysis showed that *BRAF* V600E variants, but not overall *BRAF* variants or non-V600E *BRAF* variants, were associated with inferior OS (median OS, 15.8; 95% CI, 2.7-28.9 months vs 28.3; 95% CI, 24.4-32.1 months; *P* = .04) compared with wild-type *BRAF* (eFigure 2 in Supplement 1).

### Drug Screens in Patient-Derived Organoids Carrying Different *BRAF* Variants

We selected 8 drugs that are either approved for clinical use or in clinical trials (eTable 4 in Supplement 1) and assessed their association with the viability of 6 patient-derived organoid lines harboring wild-type *BRAF* or endogenous *BRAF* variants: ICC-1 (wild-type), ICC-2 (V600E), ICC-3 (K601E), ICC-4 (D594G), ICC-5 (N581S), and ICC-6 (L597P) (eFigure 3 in Supplement 1).

Pharmacologic screens in the ICC-1 organoids revealed a slight sensitivity to lenvatinib (inhibition rate: 36%) but resistance to all 7 other drugs. The ICC-2 (V600E) organoids were sensitive to BRAF inhibition (dabrafenib, vemurafenib) as well as MEK inhibition (trametinib). The ICC-3 (K601E), ICC-4 (D594G), and ICC-5 (N581S) organoids were not sensitive to BRAF inhibition; however, they all showed sensitivity to MEK inhibition, especially by trametinib in the ICC-4 and ICC-5 organoids, which both harbored class 3 *BRAF* variants. ICC-6 (L597P) organoids exhibited broad resistance to most drugs, with only slight sensitivity to trametinib (inhibition rate: 38%) and cobimetinib (inhibition rate: 33%).

**Figure 3.**
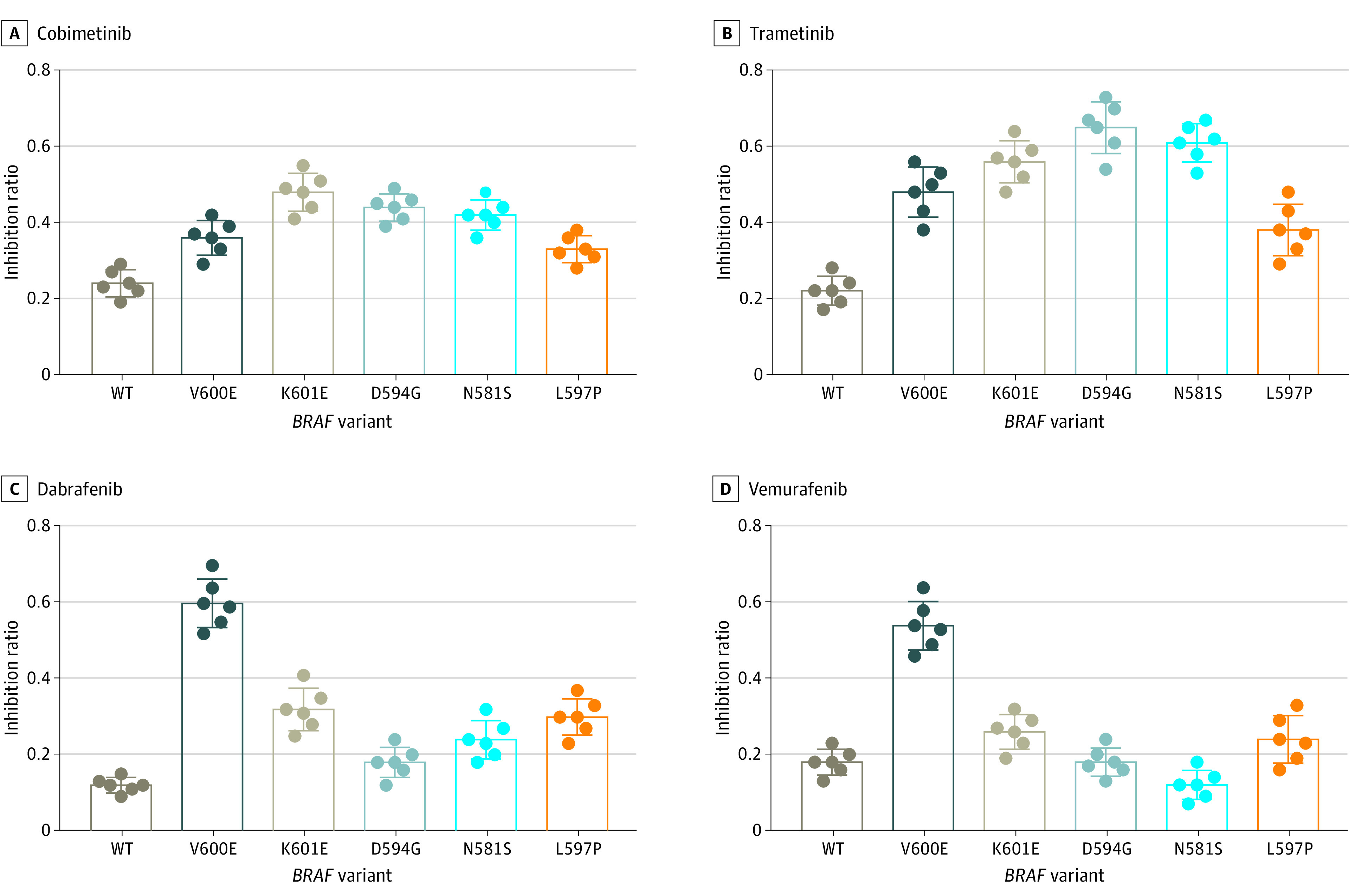
Drug Screens in Patient-Derived Organoids Comparison of BRAF with MEK inhibitors in the viability of organoids harboring different *BRAF* variants.

We further compared the association of BRAF or MEK inhibitors with the viability of organoids harboring different *BRAF* variants. The MEK inhibitor trametinib exhibited broad suppression of *BRAF* variants, especially the class 2 (K601E) and class 3 (D594G and N581S) variants. The BRAF inhibitors dabrafenib and vemurafenib showed significant suppression in *BRAF* V600E variants but had little suppression of non-V600E *BRAF* variants or wild-type *BRAF* (Figure 3).

### *BRAF* Variant Response to BRAF and MEK Inhibitors

**Figure 4.**
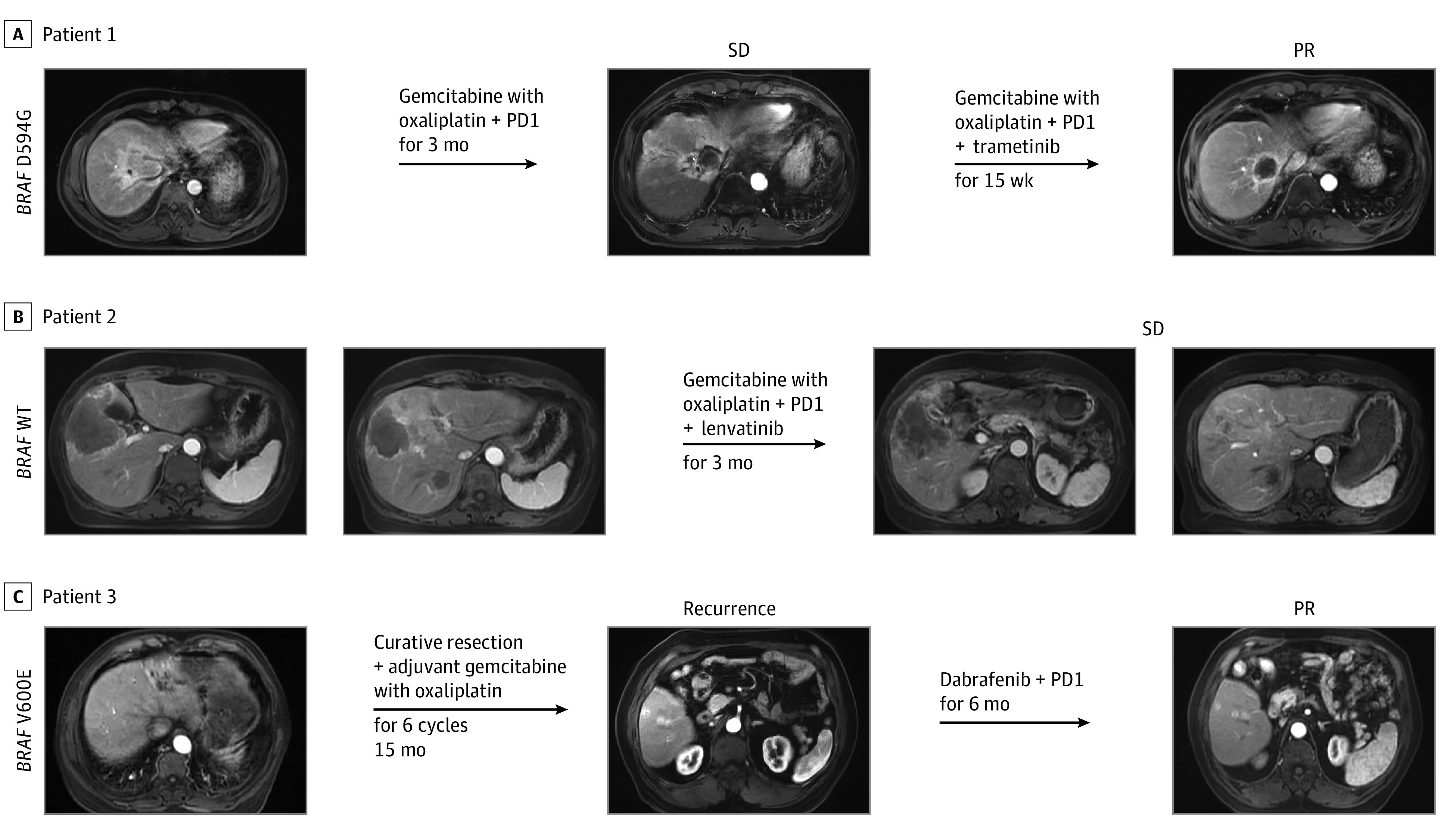
Outcomes of 3 Patients With Wild-Type (WT) *BRAF* or Variant (VT) *BRAF* Treated With BRAF or MEK Inhibitors A, Patient 1 with *BRAF* D594G variant. B, Patient 2 with wild-type (WT) *BRAF*. C, Patient 3 with *BRAF* V600E variant. PR indicates partial response; SD, stable disease.

To illustrate the importance of identifying and classifying *BRAF* variants and guide precise treatment for patients with ICC based on our drug screens in patient-derived organoids, we evaluated 3 patients with advanced-stage ICC harboring endogenous *BRAF* variants whose tumor organoids we had tested for drug response (Figure 4). Patient 1 was a man in his 50s with stage III ICC. The patient initially received gemcitabine with oxaliplatin plus PD-1 (programmed cell death 1) for 3 months and achieved a stable disease. He then underwent biopsy and sequencing that revealed a *BRAF* D594G variant. Pharmacologic screens in organoids derived from this patient (ICC-4) revealed that the organoids were most sensitive to trametinib. The patient’s treatment was switched to gemcitabine with oxaliplatin, PD-1, and trametinib, 2 mg/d, orally. This treatment provided marked clinical benefit, and the patient achieved a partial response after 15 weeks. At 12 months’ follow-up, the patient continued treatment with PD-1 and trametinib without disease progression.

Patient 2 was a woman in her 60s diagnosed with stage III lCC whose tumor harbored wild-type *BRAF*. Pharmacologic screens in organoids from this patient (ICC-1) revealed a slight sensitivity to lenvatinib, 8 mg/d, orally. The patient was subsequently treated with gemcitabine with oxaliplatin, PD-1, and lenvatinib for 3 months and achieved a stable disease.

Patient 3 was a man in his 60s diagnosed with stage II lCC. The patient received curative surgical resection and adjuvant chemotherapy for 6 cycles. Whole-exome sequencing identified a V600E genotype for *BRAF*. After 15 months, the patient experienced tumor recurrence. Biopsy and sequencing confirmed *BRAF* V600E variants in the relapsed tumor. Pharmacologic screens in organoids derived from this patient (ICC-2) revealed that the organoids were most sensitive to dabrafenib. The patient then received PD-1 and dabrafenib, 150 mg, orally twice daily for 6 months and achieved a partial response. The patient continued treatment with PD-1 and dabrafenib without disease progression until the last follow-up.

## Discussion

In this study, to our knowledge, we recruited the largest cohort of patients with ICC that has been analyzed to date. We examined *BRAF* variant subtypes and their association with patient characteristics and prognosis. The overall frequency of *BRAF* variance in our cohort was 4.2%, which is similar to that in The Cancer Genome Atlas (3.3%), Memorial Sloan Kettering 2017 (4.4%), and Memorial Sloan Kettering 2021 (6.3%) cohorts.^22,23,24^ To our knowledge, no study has investigated *BRAF* variant subtypes and classes in ICC, perhaps because of the limited sample size. The distribution of *BRAF* variant subtypes and classes in our cohort was different than that in other types of cancer, such as melanoma, thyroid cancers, CRC, and NSCLC (eFigure 4 in Supplement 1).^10^ We propose that the *BRAF* variant patterns are at least in part a product of selection during tumor initiation for an ideal level of signaling, which is shaped by variations in the function of particular *BRAF* variants, BRAF protein levels, and cellular responses to oncogenic BRAF.^25^

*BRAF* variants were not associated with survival or recurrence in the ICC cohort in this study, which is not consistent with some previous findings in cancers such as melanoma and CRC.^26,27^ We found that this inconsistency was mainly caused by the lower proportion of *BRAF* V600E variants in the ICC cohort compared with that in melanoma and CRC cohorts. Although *BRAF* V600E variants were associated with recurrence and survival in the ICC cohort, non-V600E *BRAF* variants, which accounted for most of the *BRAF* variants in the whole cohort, demonstrated no association with prognosis. A difference between *BRAF* V600E variants and non-V600E *BRAF* variants in terms of their associations with patient outcomes has also been revealed in other types of cancer.^13,28^ Although our study was single center and retrospective, the uniform standard of care and identical follow-up support the accuracy of our survival analysis.

The association between *BRAF* variant class and sensitivity to inhibitors in ICC is largely unknown. In our study, we assessed the outcomes of drug therapy using organoids and tested some of the results in patients with ICC. It has been established that BRAF inhibitors, such as dabrafenib and vemurafenib, are effective in treating tumors carrying *BRAF* V600E variants, which was also noted in ICC in our study. Strategies have been developed for the treatment of non-V600E *BRAF*-variant tumors in cancers such as CRC and NSCLC.^29,30^ In our study, we found broad differences in drug sensitivity for different *BRAF* variant classes, such as those between *BRAF* V600E variants and non-V600E *BRAF* variants. These results are inconsistent with previous results in NSCLC or CRC.^15,30^ This may be because class 1 *BRAF* variants (*BRAF* V600E variants) are RAS-independent, signal as monomers, and are sensitive to current RAF monomer inhibitors, whereas non-V600E *BRAF* variants, especially class 3 *BRAF* variants, have low or absent kinase activity and are RAS-dependent and sensitive to ERK-dependent feedback of RAS. They activate ERK by increasing their binding to RAS and require coexistent mechanisms for RAS activation.^15^ Therefore, these non-V600E *BRAF* variants may not be independent drivers; instead, they commonly act as amplifiers of the RAS signal induced by *RAS* variance, *NF1* loss, or activation of receptors, which requires further exploration.

### Limitations

This study has limitations. Our analysis is limited by the retrospective nature of the study and its exclusive focus on patients with surgically resectable disease. Given this, a degree of selection bias was largely unavoidable. Second, this study came from China, and further research is warranted for international validation.

## Conclusions

This cohort study characterized the distribution of *BRAF* variant subtypes in a large cohort of patients with ICC from China, representing what we believe to be the largest ICC cohort analyzed to date worldwide. The presence of *BRAF* V600E variants, but not non-V600E *BRAF* variants, was associated with worse survival and increased risk of recurrence. The findings suggest that identifying and classifying *BRAF* variants may help to guide precise treatment for patients with ICC.

## Notes

Supplement 1. **eMethods.** Detailed Methods
eReferences
**eTable 1.** Clinicopathologic Characteristics of Patients With Intrahepatic Cholangiocarcinoma (n=1175) **eTable 2.** 49 *BRAF* Somatic Variants Identified Across All ICC Patients **eTable 3.** Correlation Between *BRAF* Variant Status and Clinicopathologic Characteristics **eTable 4.** Drug Screening of ICC Organoid **eFigure 1.** Concise Flowchart of This Study **eFigure 2.** Association of *BRAF* Variants With Patient Outcome From Two Studies in cBioPortal (n=514) **eFigure 3.** Heatmap Shows Inhibition Ratio of 8 Drugs in Six Organoid Lines **eFigure 4.** The Prevalence and Proportion of Different *BRAF* Variants in Human Cancers

Supplement 2.
Data Sharing Statement
