# Supplementary material for: Association of BRAF Variants With Disease Characteristics, Prognosis, and Targeted Therapy Response in Intrahepatic Cholangiocarcinoma
Source: JAMA Netw Open. 2023 Mar 3;6(3):e231476. doi: 10.1001/jamanetworkopen.2023.1476 (PMC9984974; doi:10.1001/jamanetworkopen.2023.1476)
Supplement: Supplement 1. — eMethods. Detailed Methods eReferences eTable 1. Clinicopathologic Characteristics of Patients With Intrahepatic Cholangiocarcinoma (n=1175) eTable 2. 49 BRAF Somatic Variants Identified Across All ICC Patients eTable 3. Correlation Between BRAF Variant Status and Clinicopathologic Characteristics eTable 4. Drug Screening of ICC Organoid eFigure 1. Concise Flowchart of This Study eFigure 2. Association of BRAF Variants With Patient Outcome From Two Studies in cBioPortal (n=514) eFigure 3. Heatmap Shows Inhibition Ratio of 8 Drugs in Six Organoid Lines eFigure 4. The Prevalence and Proportion of Different BRAF Variants in Human Cancers [file jamanetwopen-e231476-s001.pdf]

Supplementary Online Content

Xin HY, Sun RQ, Zou JX, et al. Association of *BRAF* variants with disease characteristics, prognosis, and targeted therapy response in intrahepatic cholangiocarcinoma. *JAMA Netw Open*. 2023;6(3):e231476. doi:10.1001/jamanetworkopen.2023.1476

eMethods. Detailed Methods

eReferences

eTable 1. Clinicopathologic Characteristics of Patients With Intrahepatic Cholangiocarcinoma (n=1175)

eTable 2. 49 *BRAF* Somatic Variants Identified Across All ICC Patients

eTable 3. Correlation Between *BRAF* Variant Status and Clinicopathologic Characteristics

eTable 4. Drug Screening of ICC Organoid

eFigure 1. Concise Flowchart of This Study

eFigure 2. Association of *BRAF* Variants With Patient Outcome From Two Studies in cBioPortal (n=514)

eFigure 3. Heatmap Shows Inhibition Ratio of 8 Drugs in Six Organoid Lines

eFigure 4. The Prevalence and Proportion of Different *BRAF* Variants in Human Cancers

This supplementary material has been provided by the authors to give readers additional information about their work.

© 2023 Xin HY et al. *JAMA Network Open.*

eMethods. Detailed Methods

eMethods 1. DNA preparation, DNA capture, and sequencing

Snap frozen tissue samples from tumor and matched non-cancerous liver were obtained and embedded in OCT compound, sectioned by a cryostat, and stained by hematoxylin and eosin. We performed macrodissection to enrich the tumor fraction relative to the dominant stromal component and other normal cells. For FFPE samples, samples were cut into 5–10 consecutive 10-μm sections for laser capture microdissection (LCM). DNA was extracted using the QIAamp DNA Micro Kit (Qiagen) according to the manufacturer's protocol. Preparation of sequencing libraries and DNA capture methods were carried out according to the manufacturer's protocols.

eMethods 2. Whole-exome and targeted sequencing

For WES, snap-frozen tissue samples from 204 primary tumors and matched non-cancerous liver from 204 ICC patients were subjected to whole exome sequencing. Sequencing libraries were constructed using a modification of the KAPA Library Preparation Kit.[1] Libraries were combined into pools for solution phase hybridization using the Agilent SureDesign Human All Exon V6, and then sent for sequencing to generate 2×150 bp paired-end reads using Novaseq platform (Illumina, Inc.). For targeted sequencing, 501 frozen tumor samples from 501 ICC patients and 32 FFPE tumor samples from 32 ICC patients were subjected to targeted sequencing. We used a multiplex polymerase chain reaction-based next-generation sequencing platform, which containing 31 amplicons from genomic DNA for regions covering all coding exons in *BRAF*.

eMethods 3. Reads mapping and detection of somatic genetic alterations

Valid sequencing data was mapped to the reference human genome (UCSC hg19) using the Burrows-Wheeler Aligner (BWA)[2] software to get the original mapping results stored in BAM format. We performed local realignment of the original BAM alignment using the GATK2[3] and then marked duplicate reads using Sambamba[4].

Somatic SNVs were detected by muTect,[5] and somatic indels were detected by Strelka.[6] High-confidence somatic variants were called if the following criteria were met: (1)

© 2023 Xin HY et al. *JAMA Network Open.*

both the tumor and normal samples were covered sufficiently (≥10×) at the genomic level; (2) the variants were supported by at least 5% of the total reads in the tumor and less than 1% of the total reads in the normal tissue; (3) the variants were supported by at least three reads in the tumor. ANNOVAR[4] was performed to do annotation for the Variant Call Format obtained in the previous effort. Somatic SNVs and indels that were referenced in the 1000 Genomes Project with a minor allele frequency over 1% or located in segmental duplications were removed along with common SNPs.

eMethods 4. Sanger sequencing

All *BRAF* variants identified through WES and targeted sequencing were validated by Sanger sequencing. All coding exons of *BRAF* identified to harbor somatic variants were further screened in an additional 438 ICCs (FFPE samples). Sanger sequencing primers were designed using the Primer3 software (http://frodo.wi.mit.edu/). All variants identified in tumors were confirmed by independent PCR and Sanger sequencing in the specific tumors and their paired normal tissues to determine their somatic nature.

eMethods 5. Tumor dissociation

Fresh resected tissue was minced, rinsed with phosphate buffered saline (PBS; GIBCO), and incubated in digestion buffer on an orbital shaker at 37°C. The incubation time was dependent on the amount of collected tissue and ranged from 30 min to 90 min, lasting until the majority of cell clusters were in suspension. The digestion buffer was composed of Dulbecco's modified Eagle medium (DMEM; GIBCO) with 0.05% collagenase IV (Sigma), 0.1 mg/ml DNase I (Sigma), and 100 µg/ml Normocin (InvivoGen). After tissue digestion, DMEM containing 10% fetal bovine serum was added to the cell suspension, which was then filtered through a 70 µm Nylon cell strainer (BD Falcon) and spun for 5 min at 400*g*. The resulting pellet was washed in cold PBS twice and kept cold. During processing, 10 µl of the cell suspension was stained with Trypan Blue to determine the concentration of live cells.

eMethods 6. ICC organoid culture

The pellet was resuspended with optimized hepatobiliary tumor organoid culture medium composed of Advanced DMEM/F12 supplemented with 1% penicillin/streptomycin,

© 2023 Xin HY et al. *JAMA Network Open.*

1% GlutaMAX-I, 10 mM HEPES, 1:50 B27 supplement, 1:100 N2 supplement, 1.25 mM N-acetyl-L-cysteine, 10 mM nicotinamide, 10 nM Gastin, 50 mg/ml human EGF, 100 ng/ml human FGF10, 25 ng/ml HGF, 10 μm forskolin, 5 μm A8301, 10 μm Y27632, and 3 nM dexamethasone. Then, 5000–10000 isolated cells were mixed with cold Matrigel Basement Membrane Matrix (CORNING), and 50 μl drops of the Matrigel-cell suspension were allowed to solidify on prewarmed 24-well suspension culture plates at 37°C for 30 min. Upon complete gelation, 500 μl organoid medium was added to each well, and the plates were transferred to humidified 37°C/5% CO_2 incubators at either 2% or ambient O_2. The culture was replenished with fresh media every 3−4 days during organoid growth. Dense cultures with organoids were usually passaged with a split ratio of 1:3 every 2–3 weeks by dissociation with TrypLE Express (Gibco) and re-seeded into new Matrigel.

eMethods 7. Organoid drug screening

Information about the eight drugs used in our experiments, including drug names, targets, IC50, and source, is provided in **eTable3 in the Supplement**. 10 μL of Matrigel was dispensed into 384-well microplates and allowed to polymerize. Cells from organoids were then plated (3×10^3 per well) and cultured on the 384-well culture plates (CORNING) for 24 h, and drugs were then added to the culture medium at a final concentration of 10×10^{-6} M. After 4 days of incubation with the drugs, cell viability was assayed using CellTiter-Glo 3D Reagent (Promega) in accordance with the manufacturer's instructions. Dimethyl sulfoxide (0.1%) was used as a control. When the ratio of the average cell viability in the presence of a drug relative to that in the presence of the control was under 0.5, the drug was considered to have a significant suppressive effect.

eReferences

1. Zhou SL, Zhou ZJ, Hu ZQ, et al. Genomic sequencing identifies WNK2 as a driver in hepatocellular carcinoma and a risk factor for early recurrence. *J Hepatol.* Dec 2019;71(6):1152-1163.
2. Li H, Durbin R. Fast and accurate short read alignment with Burrows-Wheeler transform. *Bioinformatics.* Jul 15 2009;25(14):1754-1760.
3. McKenna A, Hanna M, Banks E, et al. The Genome Analysis Toolkit: a MapReduce framework for analyzing next-generation DNA sequencing data. *Genome Res.* Sep 2010;20(9):1297-1303.
4. Tarasov A, Vilella AJ, Cuppen E, Nijman IJ, Prins P. Sambamba: fast processing of NGS alignment formats. *Bioinformatics.* Jun 15 2015;31(12):2032-2034.

© 2023 Xin HY et al. *JAMA Network Open.*

5. Cibulskis K, Lawrence MS, Carter SL, et al. Sensitive detection of somatic point variants in impure and heterogeneous cancer samples. *Nat Biotechnol.* Mar 2013;31(3):213-219.
6. Saunders CT, Wong WS, Swamy S, Becq J, Murray LJ, Cheetham RK. Strelka: accurate somatic small-variant calling from sequenced tumor-normal sample pairs. *Bioinformatics.* Jul 15 2012;28(14):1811-1817.

© 2023 Xin HY et al. *JAMA Network Open.*

eTable 1. Clinicopathologic characteristics of patients with intrahepatic cholangiocarcinoma (n=1175)

Characteristics	Number (%)
Age, year (≤50 versus >50)	231/944 (19.7/80.3)
Sex (female versus male)	474/701 (40.3/59.7)
HBsAg (negative versus positive)	818/357 (69.6/30.4)
CA199(≤36 versus >36)	459/716 (39.1/60.9)
GGT,U/L (≤54 versus>54)	592/583 (50.4/49.6)
Liver cirrhosis (no versus yes)	874/301 (74.4/25.6)
Tumor size,cm(≤5 versus>5)	572/603 (48.7/51.3)
Tumor number (single versus multiple)	873/302 (74.3/25.7)
Microvascular/bile duct invasion (no versus yes)	903/272 (76.9/23.1)
Lymphatic metastasis (no versus yes)	991/184 (84.3/15.7)
Tumor encapsulation (complete versus none)	156/1019 (13.3/86.7)
Tumor differentiation (I+II versus III+IV)	505/670 (43.0/57.0)

Abreviations: GGT, gamma glutamyl transferase; CA 19-9, carbohydrate antigen 19-9.

© 2023 Xin HY et al. *JAMA Network Open.*

eTable 2. 49 *BRAF* somatic variants identified across all ICC patients

Sample	*BRAF* variant	*BRAF* variant class
ICC-18	L597V	2
ICC-61	K601E	2
ICC-117	K601E	2
ICC-140	G464V	2
ICC-165	G464E	2
ICC-190	G466E	3
ICC-220	G464V	2
ICC-226	D594G	3
ICC-340	N581I	3
ICC-345	K601E	2
ICC-411	K601E	2
ICC-420	V600E	1
ICC-436	D594A	3
ICC-442	V600E	1
ICC-454	V459M	unknown
ICC-459	R260L	unknown
ICC-474	K601E	2
ICC-501	V600E	1
ICC-533	V600E	1
ICC-540	G596R	3
ICC-581	N581S	3
ICC-636	D594G	3
ICC-656	W531X	unknown
ICC-665	N581S	3
ICC-687	V600E	1
ICC-697	V600E	1
ICC-702	G596R	3
ICC-728	K601E	2
ICC-732	L597P	unknown
ICC-747	V600E	1
ICC-767	K601E	2
ICC-783	N581S	3
ICC-826	G466V	3
ICC-836	D594G	3
ICC-837	G469A	2
ICC-882	V600E	1
ICC-883	D594G	3
ICC-896	V600E	1
ICC-897	L597P	unknown
ICC-899	A27fs	unknown
ICC-902	D594G	3
ICC-951	A35V	unknown
ICC-965	V600E	1
ICC-1013	V600E	1
ICC-1021	V600E	1
ICC-1127	V600E	1
ICC-1133	D594G	3
ICC-1142	A400T	unknown
ICC-1147	G596R	3

© 2023 Xin HY et al. *JAMA Network Open.*

eTable 3. Correlation between *BRAF* variant status and clinicopathologic characteristics

Clinicopathological indexes		No. of Patients				Test 1 *P**	Test 2 *P*†
		All patients	*BRAF* WT	*BRAF* V600E	*BRAF* non-V600E		
Age(year)	≤50	231	223	3	5	0.55	0.66#
	>50	944	903	10	31		
Sex	Female	474	448	6	20	0.06	0.56
	Male	701	678	7	16		
HBsAg	Negative	818	790	6	22	0.05	0.35
	Positive	357	336	7	14		
CA199 (U/mL)	≤20	459	439	5	15	0.80	0.84
	>20	716	687	8	21		
GGT (U/L)	≤54	592	564	6	22	0.33	0.35
	>54	583	562	7	14		
Liver cirrhosis	No	874	835	11	28	0.39	0.71#
	yes	301	291	2	8		
Tumor size(cm)	≤5	572	545	3	24	0.36	0.007
	>5	603	581	10	12		
Tumor number	Single	873	839	6	28	0.42	0.04#
	Multiple	302	287	7	8		
Vascular/bile duct invasion	absence	903	869	6	28	0.21	0.04#
	present	272	257	7	8		
Lymphatic metastasis	absence	991	954	7	30	0.08	0.06#
	present	184	172	6	6		
Tumor encapsulation	complete	156	149	1	6	0.83	0.66#
	none	1019	977	12	30		
Tumor differentiation	I+II	505	485	4	16	0.76	0.39
	III+IV	670	641	9	20		

Abbreviations: GGT, gamma glutamyl transferase; CA 19-9, carbohydrate antigen 19-9.
*Test 1 *P* value: two-sided *P* value for test of differences in factor means between *BRAF* MT v *BRAF* WT.
†Test 2 *P* value: two-sided *P* value for test of differences in factor means between *BRAF* V600E v *BRAF* non-V600E.
#Fisher's exact tests; Chi-square tests for all the other analyses
SI conversion factor: To convert GGT to μkat/L, multiply by 0.0167.

© 2023 Xin HY et al. *JAMA Network Open.*

eTable 4. Drug screening of ICC organoid

Drugs	Targets	IC50	Reference
Apatinib	VEGFR2	1nM	PMID: 21443688
	RET	13nM	
Cobimetinib	MEK1	4.2 nM	PMID: 22084396
Dabrafenib	B-Raf (V600E)	0.7 nM	PMID: 24900673
	B-Raf	5.2 nM	
	C-Raf	6.3 nM	
Lenvatinib	VEGFR2/KDR	4nM	PMID: 17943726
	VEGFR3/FLT4	5.2nM	
	VEGFR1/FLT1	22nM	
	PDGFRβ	39nM	
	FGFR1	46nM	
	PDGFRα	51nM	
	Kit	100nM	
Temuterkib	ERK1	5nM	DOI: 10.1158/1538-7445
	ERK2	5nM	
Regorafenib	RET	1.5nM	PMID: 21170960
	Raf-1	2.5nM	
	VEGFR2	4.2nM	
	Kit	7nM	
	VEGFR1	13nM	
	B-Raf (V600E)	19nM	
	PDGFRβ	22nM	
	B-Raf	28nM	
	VEGFR3	46nM	
Sunitinib	FLT3	——	PMID: 12646019
	c-Kit	——	
	PDGFRβ	2nM	
	VEGFR2	80nM	
Sorafenib	Raf-1	6nM	PMID: 15466206
	mVEGFR(Flk1)	15nM	
	mVEGFR3	20nM	
	B-Raf	22nM	
	B-Raf (V599E)	38nM	
	mPDGFRβ	57nM	
	FLT3	58nM	
	c-Kit	68nM	
	VEGFR2	90nM	
	FGFR1	580nM	
Trametinib	MEK1	0.92 nM	PMID: 21523318
	MEK2	1.8 nM	
Vemurafenib	SRMS	18 nM	PMID: 20823850
	ACK1	19 nM	
	B-Raf (V600E)	31 nM	
	C-Raf	48 nM	
	MAP4K5 (KHS1)	51 nM	
	FGR	63 nM	
	B-Raf	100 nM	
	LCK	183 nM	
	BRK	213 nM	
	NEK11	317 nM	
	BLK	547 nM	
	Lyn B	599 nM	
	YES1	604 nM	
	WNK3	877 nM	
	MNK2	1.717 μM	
	FRK (PTK5)	1.884 μM	
	CSK	2.339 μM	
	Src	2.389 μM	

© 2023 Xin HY et al. *JAMA Network Open.*

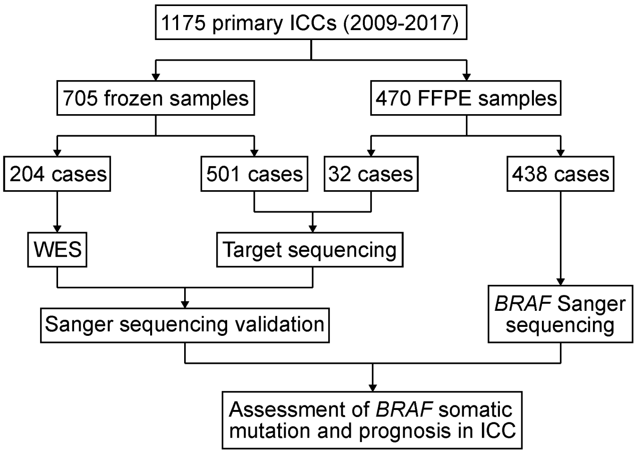

eFigure 1. Concise flowchart of this study.

© 2023 Xin HY et al. *JAMA Network Open.*

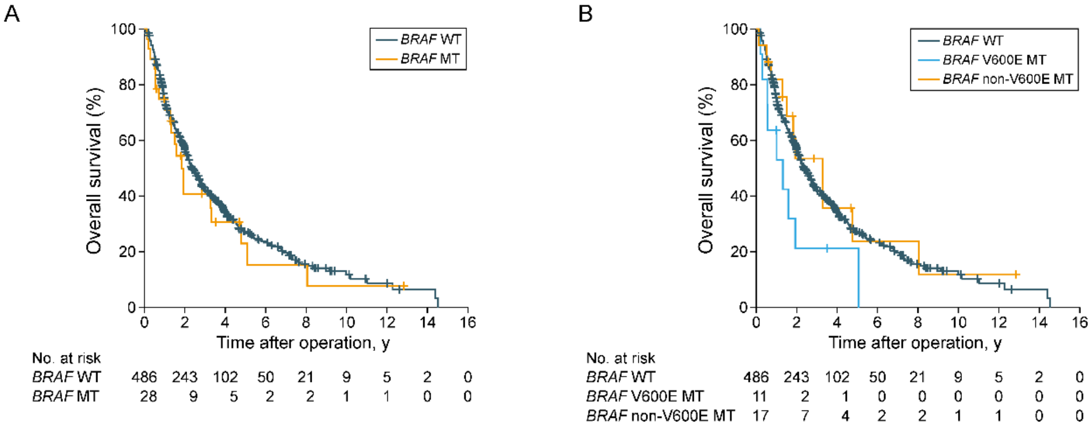

eFigure 2. Association of *BRAF* variants with patient outcome from two studies in cBioPortal (n=514).

(A) Kaplan-Meier survival analysis showing overall survival based on wt*BRAF* and mut*BRAF*. **(B)** Kaplan-Meier survival analysis showing overall survival based on wt*BRAF*, ^{V600E}*BRAF* and $^{non\text{-}V600E}$*BRAF* variants

© 2023 Xin HY et al. *JAMA Network Open.*

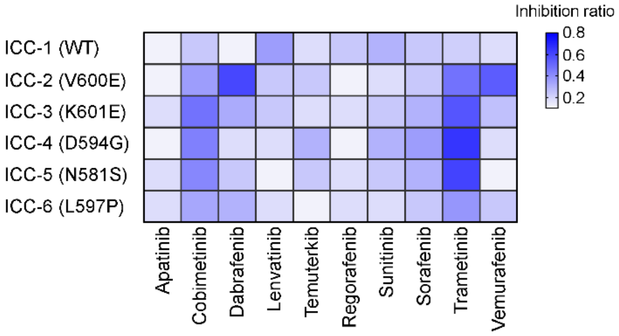

eFigure 3. Heatmap shows inhibition ratio of 8 drugs in six organoid lines. Color key from white to blue indicates relative inhibition ratio from low to high.

© 2023 Xin HY et al. *JAMA Network Open.*

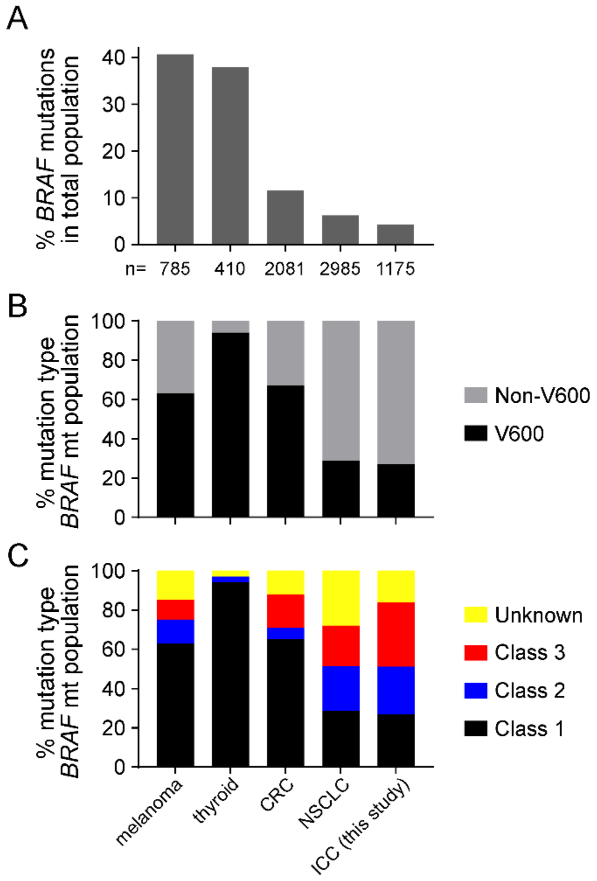

eFigure 4. The prevalence and proportion of different *BRAF* variants in human cancers. (A) Incidence of all *BRAF* variants for a given cancer type. **(B)** The proportion of V600 versus non-V600 *BRAF* variants within the total number of *BRAF* variants for a given cancer type. **(C)** The proportion of the three classes of *BRAF* variants within all *BRAF* variants for a given cancer type. CRC: colorectal cancer, NSCLC: non-small cell lung cancer.

© 2023 Xin HY et al. *JAMA Network Open.*
