# Supplementary material for: Association of BRAF Variants With Disease Characteristics, Prognosis, and Targeted Therapy Response in Intrahepatic Cholangiocarcinoma
Source: JAMA Netw Open. 2023 Mar 3;6(3):e231476. doi: 10.1001/jamanetworkopen.2023.1476 (PMC9984974; doi:10.1001/jamanetworkopen.2023.1476)
Supplement: Supplement 2. — Data Sharing Statement [file jamanetwopen-e231476-s002.pdf]

Data Sharing Statement

Xin. Association of BRAF Variances With Disease Characteristics, Prognosis, and Targeted Therapy Response in Intrahepatic Cholangiocarcinoma. *JAMA Netw Open*. Published March 03, 2023. doi:10.1001/jamanetworkopen.2023.1476

Data
Data available: No

Additional Information
Explanation for why data not available: Data described in the article will be made available upon request pending application.
